# Psychometric properties of the health literacy questionnaire tested in Vietnamese adults with chronic diseases

**DOI:** 10.1186/s12889-024-21156-7

**Published:** 2025-01-06

**Authors:** Thi Thuy Ha Dinh, Ann Bonner

**Affiliations:** 1 School of Nursing and Midwifery, Monash University, Clayton, VIC Australia; 2 School of Nursing and Midwifery, Griffith University, Brisbane, QLD Australia; 3 Kidney Health Service, Metro North Hospital and Health Service, Brisbane, QLD Australia

**Keywords:** Health literacy, Multiple chronic diseases, Psychometric property, Reliability, Confirmatory factor analysis

## Abstract

**Background:**

The Health Literacy Questionnaire (HLQ) is an increasingly used health literacy instrument that has been translated into many languages. The HLQ has 44 items and comprises 9 scales assessing the multidimensional construct of health literacy. This study reports the HLQ reliability and construct validity tested in people with chronic diseases living in Vietnam.

**Methods:**

Adults (*n* = 600) hospitalized with chronic disease in Vietnam completed the HLQ. Floor and ceiling effects, item, and scale difficulty levels were assessed. Generalized linear models with backward modeling techniques were performed to test key variables associated with each HL domain. Confirmatory factor analyses (CFA) testing nine one-factor models were fitted to test the structure of each scale, and a nine-factor model tested the hypothesized structure of the HLQ, followed by the calculation of scale reliability using Cronbach’s alpha.

**Results:**

No item had floor effects, and only eight items showed ceiling effects. Two scales that had the most difficult tasks to complete (highest difficulty level) were 8 “Ability to find good information” and 9 “Understanding enough to know what to do”. Variables associated with health literacy were education, income, age, residential area, main support persons and comorbidity index (associated with 7, 7, 4, 3, 2 and 2 out of 9 scales, respectively). Each HLQ scale demonstrated a robust unidimensional construct with all CFI ≥ 0.95, RMSEA varied from 0 to 0.07. The nine-factor CFA model demonstrated satisfactory fit indices: X2 = 5537.4, 866 df, *p* < 0.001, CFI = 0.98, NFI = 0.98, RMSEA = 0.09, 90% CI (0.093, 0.097), PCLOSE < 0.001. The highest scores were rated on scales 4 “Social support for health” and 6 “Ability to engage with healthcare providers”. The reliability of all nine scales ranged from 0.81 to 0.89.

**Conclusions:**

The Vietnamese version of the HLQ demonstrated psychometrically robust properties with high reliability and satisfactory construct validity indexes. This instrument will enable researchers, clinicians, and policymakers to assess health literacy abilities in Vietnam which could inform improvements in healthcare services and clinician practice.

**Supplementary Information:**

The online version contains supplementary material available at 10.1186/s12889-024-21156-7.

## Introduction

Together with modern architecture of healthcare system, the expansion of a variety of healthcare services, and the complexity of health insurance claims and fee structure, healthcare systems are becoming increasingly complicated for people to navigate [1]. Health literacy abilities are instrumental for a person to effectively access a health system. The World Health Organization (WHO) defined health literacy as “people’s knowledge, confidence and comfort which accumulate through daily activities and social interactions and across generations to access, understand, appraise, remember, and use information about health and healthcare, for the health and healthcare, for the health and well-being of themselves and those around them” [2]. Some countries have a national action plan on health literacy to engage organisations, healthcare professionals, policy makers and communities to improve health literacy through strategically key actions [3, 4]. In this regard, healthcare providers ought to take responsibility for filling the gaps between patients’ health literacy abilities and the complexity of health systems [5]. To do so, healthcare providers need to be aware of, familiar with, or provided with diagnostic tools to examine health literacy deficits among patients they encounter or care for.

Health literacy instruments have been evolving alongside the changing understanding of health literacy. Early instruments were used as quick screening tools to determine an individual’s functional abilities in reading words (Rapid Estimate of Adult Literacy in Medicine), reading comprehension (Test of Functional Health Literacy in Adults), or numeral calculation (Newest Vital Sign) [6]. The development of multidimensional instruments enabled a more comprehensive assessment of the different dimensions of health literacy (e.g. European Health Literacy Survey Questionnaire [7], Health Literacy Questionnaire [HLQ [8]], . Of these, the HLQ is widely used in multiple projects assessing health literacy in groups and communities [9, 10] including government-funded national surveys [11, 12]. The HLQ questionnaire was first developed in English [8] and is a 44-item instrument divided into 9 conceptually distinct scales assessing functional, communicative, social, and critical HL abilities of individuals [8]. Various language translations of the HLQ have been tested (Dutch [13], Danish [14], German [15], Urdu [16], Slovakian [17], Norwegian [18], Ghanaian [19], and Arabic [20]. These studies have involved general population [17], older adults [21], caregivers with children [19], university students [20, 22], and people with various health conditions [13, 15, 23, 24]. Although the HLQ is significantly longer than other health literacy tools mentioned above, it is a comprehensive questionnaire and can measure the real-life experiences of people on how they approach healthcare services and interact with healthcare providers [25] that cannot be assessed by other tools focusing on numeracy or comprehension. This instrument is also endorsed by the WHO for use in low and middle-income countries [26].

The Vietnamese language version of the HLQ has been translated by the instrument developers and had been used in a Vietnamese migrant sample in Australia [9]. The use of Vietnamese HLQ has been reported in several publications in Vietnam [27–29], however, the psychometric properties have not been tested. The availability of the HLQ in Vietnamese, along with evidence of its psychometric reliability, is essential for advancing health literacy research and practice in Vietnam, where the awareness in this issue is still developing. Individuals with lower health literacy tend to delay seeking healthcare and misinterpret health information which can lead to misunderstanding of medical instructions, and so often experience more emergency department visits [30] and poorer healthcare outcomes [31]. Understanding about health literacy of individuals is important to assist healthcare professionals tailoring support to them and inform policy at national and organizational levels. Evidence for the psychometric properties of translated instruments is necessary to ensure all scales are reliable in native-speaking participants who are interacting with healthcare professionals and health system in that region. This study used data from a large survey of people with chronic diseases residing in Vietnam to determine the validity, reliability, and factor structure of the Vietnamese HLQ.

## Methods

### Study design

This study used secondary data from a cross-sectional survey to test the psychometric properties of the HLQ. The research questions driving this paper were: (1) How difficult is it to score each individual item; (2) Are the scales internally consistent; (3) Is each scale unidimensional, and (4) Does the original 9-domain model of the HLQ fit with data derived from people residing in Vietnam?

### Setting, participants and sample size

Participants were recruited from hospitalized patients in three inpatient wards (cardiology, nephrology, diabetes) in a large tertiary hospital in Hanoi, Vietnam from January to June 2019. This hospital receives referrals from all provincial hospitals in the north of Vietnam, representing the diversity of geographic residence and socioeconomic background amongst patients.

Eligible participants were recruited using a convenience sampling method across all study sites until the required sample size was reached. A medical doctor in each ward screened patients and referred those who met the selection criteria to the research team. Participants were invited to complete the HLQ if they were adults (aged ≥ 18), had been diagnosed with at least one chronic condition (e.g., heart failure, diabetes, or chronic kidney disease), and agreed to participate. Individuals with critical illnesses, cognitive impairments identified by the treating medical doctor, or those unable to communicate verbally were excluded from the study. Research assistants obtained consent and helped participants complete the questionnaires, reading aloud each question when needed. Four research assistants were final-year undergraduate nursing students, trained by the research team to approach patients, obtain consent, and assist with responses. Completion of questionnaires took approximately 20–30 min.

Sample size was calculated primarily to test a structural equation model with 44 variables following the rule-of-thumb ratio requiring at least 10 participants per variable [32]. To account for potential missing data, an additional 20% was added, resulting in a minimum required sample size of 528 participants. This sample size was also sufficient for the psychometric evaluation of the HLQ, adhering to the guideline of having at least 10 participants per item.

### Measures

Participants were asked to complete a demographic questionnaire and the Vietnamese version of the HLQ. The demographic data collected included age, gender, residential area, highest level of education, current occupation, monthly household income in Vietnamese dong (VND), health insurance status, and main support person. Additionally, medical data were extracted from participants’ medical records to calculate the Age-adjusted Charlson Comorbidity Index (ACCI). The ACCI was determined by summing scores assigned for age and the presence of chronic diseases or conditions. Age was scored as 1 point for every decade starting at age of 40, while chronic diseases or conditions were assigned scores ranging from 1, 2, 3 or 6 based on their associated severity [33].

The HLQ comprises 9 conceptually distinct scales that evaluate individuals’ functional, communicative, social, and critical HL capabilities [8]. Scales 1–5 assess the level of agreement with statements using a 4-point Likert scale (1 = strongly disagree to 4 = strongly agree) across the following areas (1) healthcare provider support, (2) having sufficient information, (3) actively managing health, (4) having social support, (5) appraisal of health information. Scales 6–9 measure the difficulty of performing specific tasks using a 5-point Likert scale (1 = cannot do or always difficult to 5 = always easy) to assess scales regarding (6) actively engage in health system, (7) navigate the health system, (8) find good health information, and (9) understand health information. The score for each scale is calculated by averaging the total item scores, with no overall score computed for the entire instrument. The original HLQ validation study reported composite reliability for each scale ranging from 0.77 to 0.9 [8].

The Vietnamese version of the HLQ was translated by the instrument developers, who also prepared versions in other languages. The developers explicitly outlined the rigorous translation methods they employed, which included forward and backward translation process. The translation team comprised of bilingual healthcare professionals, patients, and native-speaking local consumers. Central to this method is the principle of translation integrity, requiring the translation team to adhere to each item’s intent description to ensure sematic equivalence between English and translated versions [34]. This questionnaire has been used in a Vietnamese sample in Australia [9] and in Vietnam [27–29]. We obtained a license from the instrument developers to use the Vietnamese version of the HLQ.

### Data analysis

Data were entered into IBM SPSS version 29^®^ and examined for missing values, outliers, and normality. Floor and ceiling effects were evaluated for all items and were considered present if more than 15% of participants scored either the lowest (score = 1) or the highest possible score (score = 4 for scales 1–5 or 5 for scales 6–9). Item difficulty was analysed using descriptive statistics consistent with previous HLQ studies [8, 14, 23]. For scales 1–5 (part 1 of the questionnaire), item difficulty was determined as the proportion of responses scored “disagree” or “strongly disagree” compared to those scored “agree” or “strongly agree”. For scales 6–9 (part 2 of the questionnaire), item difficulty was calculated as the proportion of responses indicating “cannot do or always difficult”, “usually difficult”, or “sometimes difficult” compared to those indicating “usually easy” or “always easy”. Item difficulty reflects how challenging it was for participants to achieve high scores, with higher difficulty indicating items that were harder for participants to score highly on.

Descriptive analyses were conducted to describe the sample, using number and frequencies for categorical variables; and mean with standard deviations for continuous variables. The ACCI was categorized into 2 groups based on the mean score (ACCI = 6.68): those with an ACCI score < 6; and those with a score ≥ 6. Means, standard deviations, and effect sizes for differences in HLQ domain scores across demographic groups were calculated. Cohen’s *d* was used to measure effect size, with thresholds interpreted as small (0.2–0.49), medium (0.5–0.79), and large (≥ 0.8). HLQ scales were standardized, with means ranging from 1 to 4 (scales 1–5) and from 1 to 5 (scales 6–9). Multivariate analyses were performed using generalized linear models with a backward elimination modelling technique to identify key variables associated with each HL scale. The tested variables were gender (male, female), age group (< 65, ≥ 65), level of education (up to year 12 education, a degree or higher education), household income (≤ 5, 5–10, ≥ 10 million VND), marital status (married, single/divorced/widowed), residential area (urban settings, rural areas), main support person (spouse/children, others), and ACCI index (< 6, ≥ 6). These variables were selected for inclusion in the multivariate models based on their significant correlation with HL scales (*p* ≤ 0.05). The backward elimination process was conducted iteratively, removing the variable with the highest non-significant p value (> 0.05) at each step. This procedure continued until all remaining variables in the final model were statistically significant.

Given that hypothesized constructs were specified a priori, confirmatory factor analysis (CFA) was used to test nine one-factor models demonstrating the unidimensional construct of each scale using diagonally-weighted least squares (DWLS) in JASP software (version 0.18.3) [35]. A correlated 9-factor model was performed to test the overall HLQ structure (8). Goodness-of-fit criteria were comparative fit index (CFI) ≥ 0.95, Normed Fit Index (NFI) ≥ 0.95, that the test for root-mean-square error of approximation (RMSEA) ≤ 0.05 was not significant (or PCLOSE ≥ 0.05) or the higher bound of 90% CI of RMSEA falls into the range of [0.06, 0.08] or lower [36]. When a CFA model did not have an adequate fit to the data, modification indices were used to inform potential improvements to fit indices, with the principle that the addition or deletion of covariance between measurement errors needed to have substantive meaning, and by doing so improved the model’s goodness-of-fit [32].

## Results

### Demographic and health characteristics

A total of 600 participants were recruited, with a mean age of 61 years (± 15.3), and 54% were male. The majority were married (84%), most had an education level of Year 12 or below (82.4%), and approximately half resided in rural or remote areas (56.4%). Nearly two-thirds (62.5%) reported a monthly family income of 5 million VND or less (~ US$215). All participants had multiple chronic diseases, with the most common being hypertension (89%), diabetes (62%), CKD (61%) and heart failure (28%). The mean ACCI was 6.68 (SD = 2.1) and 71.3% of participants had an ACCI of 6 or higher. Table 1 provides an overview of the demographic and health characteristics.

**Table 1:** Participants’ characteristics (*n* = 600)

Characteristics	Number	Percentage
**Age group**		
< 65 years	307	51.2%
≥ 65 years	293	48.8%
**Gender**		
Female	274	45.7%
Male	326	54.3%
**Marital status**		
Married	502	83.7%
Divorced/Widowed/Single	98	16.3%
**Residential area**		
City/town	262	43.7%
Rural/remote/mountainous	338	56.3%
**Level of education**		
Up to year 12	494	82.3%
VET/University/Higher degree	106	17.7%
**Monthly income**		
≤ 5 million VND ($US215)	395	65.8%
5–10 million VND ($US215-430)	128	21.3%
≥ 10 million VND ($US430)	77	12.8%
**Main support person**		
Spouse/children	488	81.3%
Others	112	18.7%
**Health insurance**		
Yes	582	97%
No	18	3.0%
**Chronic disease diagnosis^**		
Heart failure	170	28.3%
Hypertension	537	89.5%
Diabetes	375	62.5%
Chronic kidney disease	367	61.2%
**On dialysis**		
Yes	146	24.3%
No	454	75.7%
**Age-adjusted Comorbidity index (ACCI)**		
< 6	172	28.7%
≥ 6	428	71.3%
**Age**, mean, SD	61.54 (15.3)	
**ACCI**, mean, SD		6.68 (2.1)

Note. ACCI: Age adjusted Charlson Comorbidity Index. VET: vocational education and training, typically 2–3 years after high school. VND: Vietnamese Dong. USD: US dollar. SD: standard deviation. ^Diagnosis adds to > 100% due to comorbidity

### Floor and ceiling effects

No items demonstrated a floor effect, which items ranging from 0 to 0.13. Across the nine scales, 8 items demonstrated ceiling effects (0.16–0.44); all of these items were in scales 3 “Actively managing your health” (3 items 1.6, 1.13, 1.21) and 4 “Social support for health” (all 5 items in this scale).

### Difficulty level

Scales 3 “Actively managing your health” and 4 “Social support for health” showed the lowest difficulty, indicating these were the easiest for participants to score highly, as the average difficulty levels were 0.27 and 0.07 respectively. The most difficult scales were 8 “Ability to find good information” (average item difficulty 1.16) and 9 “Understanding enough to know what to do” (average item difficulty 1.24).

In scales 1–5 with 4- response options (strongly disagree – strongly agree), the easiest items to score highly were items HLQ 1.3, 1.5, 1.11 which all belonged to scale 4 (Social support for health). The hardest items were items HLQ 1.2 “I have at least one healthcare provider who….” and HLQ 1.12 “I always compare health information from….” (the difficulty level of 1.03 and 1.13, respectively).

In scales 6–9 with 5-response options (cannot do, always easy), items HLQ 2.2, 2.9. 2.21 were the easiest to score (item difficulty of 0.41, 0.39, 0.31 respectively); while items HLQ 2.5, 2.12, 2.17 (Confidently fill medical forms in the correct…; Read and understand written health…; Read and understand all the information on…) were the most difficult (item difficulty of 1.53, 1.63 and 2.33).

### Health literacy profiles and associations

Scales 1–5 assessed participants’ perceptions of healthcare providers’ support, availability of health information, their abilities to actively manage their health, social support for managing health, and abilities to evaluate health information. Among these, Scale 4 *Social support for health* had the highest mean score (mean = 3.24, SD = 0.42). Other scales scored lower, including scale 1 *Feeling understood and supported by healthcare provider support* (mean = 2.61, SD = 0.51), scale 5 *Appraisal of health information* (mean = 2.62, SD = 0.48), scale 2 *Having sufficient information to manage my health* (mean = 2.71, SD = 0.5).

Scales 6–9 evaluated the level of difficulty participants experienced in engaging with healthcare providers, navigating the healthcare system, finding reliable information, and understanding health information. Two scales in this group had lower mean scores, suggesting participants found these tasks somewhat challenging: Scale 8 *Ability to find good health information* (mean = 3.29, SD = 0.76) and Scale 9 *Understanding health information well enough to know what to do* (mean = 3.29, SD = 0.71). The highest score in this group was for Scale 6 *Ability to actively engage with healthcare provider* (mean = 3.65, SD = 0.59). The mean score of each HLQ scale is summarised in Table 2. Further details on the health literacy profiles of this sample have been published elsewhere [28].

**Table 2:** Health literacy questionnaire scales’ mean scores

	Number of items	Mean (Standard deviation)	95% Confidence Interval
Range 1 (lowest) − 4 (highest)			
1. Feeling understood and supported by healthcare providers	4	2.61 (0.51)	2.57, 2.65
2. Having sufficient information to manage my health	4	2.71 (0.50)	2.67, 2.75
3. Actively managing my health	5	2.94 (0.44)	2.91, 2.98
4. Social support for health	5	3.24 (0.42)	3.21, 3.28
5. Appraisal of health information	5	2.62 (0.48)	2.58, 2.66
Range 1 (lowest) − 5 (highest)			
6. Ability to actively engage with healthcare providers	5	3.65 (0.59)	3.61, 3.70
7. Navigating the healthcare system	5	3.61 (0.59)	3.56, 3.65
8. Ability to find good health information	5	3.29 (0.76)	3.23, 3.35
9. Understanding health information well enough to know what to do	5	3.29 (0.71)	3.24, 3.35

Generalized linear models examining the associations between demographic variables and each HLQ scale revealed that education was significantly associated with 7 of the 9 scales (scales 1, 3, 5, 6, 7, 8, 9). Similarly, income was linked to 7 scales (scales 2, 4, 5, 6, 7, 8, 9); followed by age (scales 6, 7, 8, 9); residential area (scales 1, 2, 4), main support persons (scales 1, 4) and comorbidity index (scales 5, 8). Mean scores for groups with significantly higher health literacy, adjusted for confounders, such as education and income, are presented in Table 3. Additional details of of the models parameters are provided in the Supplemental Tables 1a, b. Overall, the effect sizes indicated a very small to small difference in HL domains across demographic characteristics, except for some medium to large ESs. The largest effect size (ES = 0.86) was observed in Scale 8 *Ability to find good health information*), attributed to family income (see Supplemental Tables 2a, b). Further details of these models have been published elsewhere [27].

**Table 3:** Generalised linear models testing factors associated with higher levels of health literacy

Associated factor/ HLQ scales	Education: Vocational training/university (*n* = 106) Mean (95% CI)	Income: ≥ 10 million VND/month (*n* = 77) Mean (95% CI)	Age: < 65 years (*n* = 307) Mean (95% CI)	Residence: Living in urban area (*n* = 262) Mean (95% CI)	Main support person: Spouse/Children (*n* = 488) Mean (95% CI)	ACCI: <6 (*n* = 172) Mean (95% CI)
Range 1 ‘strongly disagree’ – 4 ‘strongly agree’						
1. Feeling understood and supported by HCPs	2.74 (2.64, 2.84)**			2.69 (2.62, 2.77)*	2.70 (2.65, 2.76)*	
2.Having sufficient information to manage my health		2.89 (2.78, 3.00)**		2.83 (2.77, 2.90)*		
3.Actively managing my health	3.04 (2.96, 3.13)*					
4.Social support for health		3.39 (3.30, 3.48)**		3.31 (3.25, 3.37)**	3.35 (3.30, 3.39)**	
5.Appraisal of health information	2.86 (2.77, 2.96)**	2.82 (2.72, 2.93)*				2.80 (2.72, 2.88)*
Range 1 ‘always difficult’ – 5 ‘always easy’						
6.Ability to actively engage with HCPs	3.91 (3.80, 4.01)**	3.94 (3.81, 4.07)**	3.87 (3.79, 3.94)**			
7.Navigating the healthcare system	3.92 (3.81, 4.03)**	3.90 (3.77, 4.03)**	3.84 (3.76, 3.91)**			
8.Ability to find good health information	3.73 (3.59, 3.87)**	3.77 (3.61, 3.93)**	3.64 (3.54, 3.73)**			3.67 (3.54, 3.80)*
9.Understanding health information well enough to know what to do	3.65 (3.52, 3.78)**	3.68 (3.52, 3.83)**	3.64 (3.55, 3.73)**			

*p < 0.05 **p < 0.01

*Comparison groups*: Education: Up to year 12, Family income < 10 million VND per month, Age: < 65, Residence: Living in rural/remote area, Main support person: Others (not spouse or children), ACCI ≥ 6

Abbreviations: ACCI: Age-adjusted Charlson Comorbidity Index, CI: Confidence Interval, HLQ: Health Literacy Questionnaire, HCPs: Healthcare practitioners

Confounders adjusted in each model were Education, Income, Age group, Residence, Main support person, Comorbidity Index

### Psychometric testing of HLQ

The psychometric properties of the HLQ are shown in Table 4. The Cronbach’s alpha reliability was ≥ 0.8 for all scales. The model fits for all scales were generally good that all CFIs ≥ 0.95, RMSEAs varied from 0 to 0.07. For each scale, factor loadings were satisfactory to high, with 40 out of 44 items showing factor loadings of ≥ 0.60 (ranging from 0.60 to 0.94). Four items had a low factor loading: item 1.17 “I have the healthcare providers I need to help me…” (0.59); item 1.20 “I ask healthcare providers about…” (0.57); item 2.9 “Accurately follow the instructions…” (0.43) and item 2.21 “Understand what healthcare providers…” (0.55). Interestingly, all 4 items related to the communication between the person and healthcare providers.

**Table 4:** Factor loadings, model fit indexes and Cronbach’s alpha of each of nine one-factor models testing each Health Literary Questionnaire scale

Scale/item	Factor loading	Fit indexes	Coefficient alpha
Scale 1: Healthcare provider support			
HLQ 1.2	0.91	X2 = 5.63, 2 df, *p* = 0.06, CFI = 1.0, NFI = 0.99, RMSEA = 0.05, 90% CI (0.00, 0.11), PCLOSE = 0.35.	0.83
HLQ 1.8	0.93		
HLQ 1.17	0.59		
HLQ 1.22	0.84		
Scale 2: Having sufficient information			
HLQ 1.1	0.73	X2 = 7.45, 2 df, *p* = 0.02, CFI = 0.99, NFI = 0.99, RMSEA = 0.07 (90% CI 0.02, 0.12), PCLOSE = 0.22.	0.85
HLQ 1.10	0.87		
HLQ 1.14	0.87		
HLQ 1.23	0.87		
Scale 3: Actively managing my health			
HLQ 1.6	0.82	X2 = 82.1, 5 df, p = < 0.001, CFI = 0.99, NFI = 0.99, RMSEA = 0.16 (90% CI 0.13, 0.19), PCLOSE < 0.001. The MI (= 77.3) suggested a correlated error between items 1.9 and 1.18 is required (correlation coefficient = 0.3, *p* < 0.001), that resulted in improved model fit indexes (X2 = 4.61, 4 df, *p* = 0.33, CFI = 1.0, NFI = 0.99, RMSEA = 0.02 (90% CI 0.00, 0.07), PCLOSE = 0.83.	0.83
HLQ 1.9	0.71		
HLQ 1.13	0.89		
HLQ 1.18	0.68		
HLQ 1.21	0.76		
Scale 4: Social support for health			
HLQ 1.3	0.79	X2 = 43.1, 5 df, *p* < 0.001, CFI = 0.99, NFI = 0.99, RMSEA = 0.11 (90% CI 0.08, 0.14), PCLOSE < 0.001. The MI (= 27.0) suggested a correlated error between items 1.5 and 1.15 is required (correlation coefficient = 0.24, *p* < 0.001), that resulted in improved model fit indexes (X2 = 16.1, 4 df, *p* = 0.003, CFI = 0.99, NFI = 0.99, RMSEA = 0.07 (90% CI 0.04, 0.11), PCLOSE = 0.14.	0.81
HLQ 1.5	0.62		
HLQ 1.11	0.87		
HLQ 1.15	0.75		
HLQ 1.19	0.87		
Scale 5: Appraisal of health information			
HLQ 1.4	0.88	X2 = 47.9, 5 df, *p* < 0.001, CFI = 0.99, NFI = 0.99, RMSEA = 0.12, 90% CI (0.09, 0.15), PCLOSE < 0.001. The MI (= 36.2) requested a correlated error is required between items 1.16 and 1.20 (correlation coefficient = 0.20, *p* < 0.001), resulted in improved model fit (X2 = 11.7, 4 df, *p* = 0.02, CFI = 0.99, NFI = 0.99, RMSEA = 0.06, 90% CI (0.02, 0.09), PCLOSE = 0.33	0.84
HLQ 1.7	0.84		
HLQ 1.12	0.92		
HLQ 1.16	0.70		
HLQ 1.20	0.57		
Scale 6: Ability to actively engage with HCPs			
HLQ 2.2	0.75	X2 = 12.7, 5 df, *p* = 0.03, CFI = 0.99, NFI = 0.99, RMSEA = 0.05, 90% CI (0.02, 0.09), PCLOSE = 0.43.	0.89
HLQ 2.4	0.86		
HLQ 2.7	0.93		
HLQ 2.15	0.88		
HLQ 2.20	0.84		
Scale 7: Navigating the healthcare system			
HLQ 2.1	0.63	X2 = 74.1, 9 df, *p* < 0.001, CFI = 0.99, NFI = 0.99, RMESA = 0.11, 90% CI (0.09, 0.13), PCLOSE < 0.001. The MI (= 41.1) suggested a correlated error is required between items 2.16 and 2.19 (correlation coefficient = 0.15, *p* < 0.001) resulted in improved model fit (X2 = 33.3, 8 df, *p* < 0.001, CFI = 0.99, NFI = 0.99, RMSEA = 0.07, 90% CI (0.05, 0.09), PCLOSE = 0.06).	0.89
HLQ 2.8	0.86		
HLQ 2.11	0.82		
HLQ 2.13	0.92		
HLQ 2.16	0.79		
HLQ 2.19	0.84		
Scale 8: Ability to find good health information			
HLQ 2.3	0.86	X2 = 126.3, 5 df, *p* < 0.001, CFI = 0.99, NFI = 0.99, RMSEA = 0.20, 90% CI (0.17, 0.23), PCLOSE < 0.001. The MI (= 122.7) suggested a correlated error is required between items 2.10 and 2.14 (correlation coefficient = 0.29, *p* < 0.001) resulted in X2 = 3.45, 4 df, *p* = 0.48, CFI = 1.0, NFI = 1.0, RMSEA = 0.00, 90% CI (0.00, 0.06), PCLOSE = 0.91.	0.89
HLQ 2.6	0.93		
HLQ 2.10	0.62		
HLQ 2.14	0.73		
HLQ 2.18	0.95		
Scale 9: Understand health information			
HLQ 2.5	0.87	X2 = 118.0, 5 df, *p* < 0.001, CFI = 0.99, NFI = 0.99, RMSEA = 0.19, 90% CI (0.16, 0.22), PCLOSE < 0.001. The MI (= 115.6) suggested a correlated error is required between items 2.9 and 2.21 (correlation coefficient = 0.38, *p* < 0.001) resulted in improved model fit (X2 = 2.18, 4 df, *p* = 0.70, CFI = 1.0, NFI = 1.0, RMSEA = 0.00, 90% CI (0.00, 0.05), PCLOSE = 0.96).	0.84
HLQ 2.9	0.43		
HLQ 2.12	0.96		
HLQ 2.17	0.91		
HLQ 2.21	0.55		

The largest modification index in each of six one-factor CFA models was used to guide the addition of a correlated residual that resulted in perfect fit models (HLQ 3 - items 1.9 and 1.18; HLQ 4 - items 1.5 and 1.15; HLQ 5 - items 1.16 and 1.20; HLQ 7 - items 2.16, 2.19; HLQ8 - items 2.10 and 2.14; HLQ 9 - items 2.9 and 2.21).

A correlated nine-factor CFA model with no cross-loadings and no correlated residuals showed fit indices: X2 = 5537.4, 866 df, *p* < 0.001, CFI = 0.98, NFI = 0.98, RMSEA = 0.09, 90% CI (0.093, 0.097), PCLOSE < 0.001, among them CFI and NFI indicated a good model fit. The item factor loading results are presented in Supplementary Table 3. The inter-factor correlations between scales in Part 1 were 0.42–0.79. Low-moderate correlations were found between scales in Part 1 and Part 2 (ranging from 0.22 to 0.67). The inter-factor correlations between scales in Part 2 were high from 0.76 to 0.96. The highest correlations were found between scale 6 “Actively engage with HCPs” and scale 7 “Navigating the healthcare system” (*r* = 0.96); between scale 8 “Ability to find good health information” and scale 9 “Understand health information enough to know what to do” (*r* = 0.90) (see Supplemental Table 4).

## Discussion

When instruments are translated into other languages, it is important to evaluate the psychometric properties in the targeting population. This study tested the psychometric properties of the Vietnamese version of the HLQ in people with chronic diseases residing in Vietnam. Overall, the CFA model showed acceptable fit indices of a highly restrictive nine-factor construct that supported the HLQ’s hypothesized factor structure, and all nine scales were valid and had a strong composite reliability coefficient (all > 0.8). We also found some patterns of responses to HLQ scales due to different demographic profiles.

At the item level, all items showed no floor effects, which indicated that no items were too hard to complete. Only eight items demonstrated a ceiling effect (> 15% participants rated it with the highest score), and interestingly, these items were all located in two scales 3 “Actively managing your health” and 4 “Social support for health”, indicating that many participants had the ability to manage their health or felt supported by others to do so. The items that were most difficult to score highly were related to having a regular healthcare provider (item 1.2), appraising health information (item 1.12), and reading and understanding written health information (items 2.2, 2.9, 2.21). The underlying reasons making these items more difficult for those in Vietnam are rooted in the fact that general practitioners (or family doctors) are just an emerging part of the healthcare system in Vietnam. For those with chronic disease, they need to attend larger acute care hospitals for routine examination, diagnosis, treatment, and even for repeat prescriptions. This means that most people do not have a regular doctor (or nurse). Additionally, many participants had lower school education that likely limited their ability to read and understand health information (assuming that they had been provided with this information). A paradigm shift from placing the responsibility of understanding information on patients to that of an organization where systems and policies support healthcare providers to effectively communicate with patients is required [37].

The item/scale difficulty level we obtained seems to be different from other language testing studies. We found the scales with the highest difficulty scores were 8 and 9 (ability to find good health information, ability to understand information well enough). This varies between language versions, for instance, the Danish scales 2 and 4 were the easiest and scales 5 and 7 the hardest [14]. It is likely that several factors (e.g. socioeconomic, education, culture, health systems and clinician practice) influence health literacy abilities so that comparing health literacy scores between different countries should be done with caution. The effect sizes also confirmed the results of multivariate models that socioeconomical status, mostly income, can be an important indicator of differences in health literacy, as well as inequity in healthcare access. The scrutiny of items that had correlated residuals revealed either the similarity in the original wording, as well as the Vietnamese translation wording, for example “Get health information about health so you are…” (item 2.10) and “Get health information in words you…” (item 2.14), or two items could refer to similar actions, for example, a person could “know how to find out if the health information.” (item 1.16) by “ask healthcare providers about….” (item 1.20), despite that these words and actions differ in level of cognitive ability and health literacy. In disseminating this questionnaire, data collectors ought to remind people to read each sentence slowly and if possible, read them aloud and explain the semantic meaning and nuances where necessary to avoid similar responses to different statements.

This study found a high correlation between scale 6 “Actively engage with HCPs” and scale 7 “Navigating the healthcare system”, between scale 8 “Ability to find good health information” and scale 9 “Understand health information enough to know what to do”. Other HLQ validation studies have also found similar high correlations between these scales (see ref numbers 8, 15, 16, 24). Probably, these scales measure closely related concepts which require related skills. Elsworth et al. (2022) has tested several alternative model specifications, demonstrated that there could be a general factor that might be general health literacy underlying each HLQ item that could explain for the high inter-factor correlations, and therefore discriminant validity of the HLQ scales needs further investigation [38].

This study found clear patterns of lower health literacy abilities on many scales across several subgroups (age > 65 years, lower education, lower income). Low health literacy in any domain might hinder an individual’s ability to successfully adhere to treatment regimens and self-management which is crucial to slow the progression of chronic diseases and to avoid long-term consequences (e.g. increased hospitalizations, dialysis, increased mortality). Clearly reading ability is the foundation of health literacy, and it is closely associated with years of education. This study also reported lower health literacy scores for those living rurally. No other HLQ testing studies have found this difference. Nonetheless, our results indicate that the HLQ can detect meaningful differences in health literacy abilities across various demographic groups.

### Limitations

The questionnaires were self-rated which might have reporting bias as participants could have overrated or underestimated their health literacy abilities. In addition, as all participants had chronic diseases which necessitated more frequent interactions with healthcare providers and services, these results may not be generalizable to the healthy population, or people living in other regions in Vietnam. The use of a convenience sampling technique could also result in a skewed sample, as individuals with higher health literacy were more likely to participate, that represents a limitation of this study.

### Further research

Further HLQ testing should include various settings including primary healthcare and communities to provide health literacy profiles in more diverse demographic groups and test the discriminant validity of HLQ scales.

## Conclusion

This study demonstrated high reliability and robust construct validity of the HLQ, which support the use of this instrument to assess health literacy in Vietnamese speaking populations. This study provided insight into the areas that adults with chronic diseases might have difficulties in accessing, obtaining, and using health information. The understanding of health literacy profiles of those with chronic diseases will help policymakers to improve healthcare access through enhancing healthcare providers practices in Vietnam. Approaches for enhancing health literacy ought to address the unique needs of individuals with heightened focus on underserved socioeconomic groups.

## Electronic supplementary material

Below is the link to the electronic supplementary material.

Supplementary Material 1

## Data Availability

The dataset generated and analysed during this current study is not publicly available due to sharing data was not part of this study’s ethics approval. The corresponding author should be contacted if there is any request relating to this study data.

## Abbreviations

WHO: World Health Organization
HLQ: Health Literacy Questionnaire
VND: Vietnam dong
ACCI: Age-adjusted Charlson Comorbidity Index
CFA: Confirmatory factor analysis
X2/df: Chi-square divided by degree of freedom
GFI: Goodness-of-fit index
AGFI: Adjusted goodness-of-fit index
RMSEA: Root mean square error of approximation
CFI: Comparative Fit index
CI: Confidence interval
SD: Standard deviation
HCP: Healthcare providers

## References

[1] Calder R, Dunkin R, Rochford C, Nichols T. Australian health services: too complex to navigate. A review of the national reviews of Australia’s health service arrangements. Policy Issues Paper No. 1. Australian Health Policy Collaboration; 2019.

[2] World Health Organization. Health literacy development for the prevention and control of noncommunicable diseases: volume 1. Overview. Geneva: WHO; 2022.

[3] US Department of Health and Human Services, Office of Disease Prevention and Health Promotion. National action plan to improve health literacy. 2010. Washington, DC.

[4] Australian Commission on Safety and Quality in Health Care. Health literacy: taking action to improve safety and quality. Sydney: ACSQHC, Pg 21.

[5] Agency for Healthcare Research and Quality. AHRQ Health Literacy Universal Precautions Toolkit. Retrieved from https://www.ahrq.gov/health-literacy/improve/precautions/index.html

[6] Urstad KH, Andersen MH, Larsen MH, Borge CR, Helseth S, Wahl AK. Definitions and measurement of health literacy in health and medicine research: a systematic review. BMJ Open. 2022;12(2):e056294. 35165112 10.1136/bmjopen-2021-056294 PMC8845180

[7] Sørensen K, Van den Broucke S, Pelikan JM, Fullam J, Doyle G, Slonska Z, et al. Measuring health literacy in populations: illuminating the design and development process of the European Health Literacy Survey Questionnaire (HLS-EU-Q). BMC Public Health. 2013;13:948. 24112855 10.1186/1471-2458-13-948 PMC4016258

[8] Osborne RH, Batterham RW, Elsworth GR, Hawkins M, Buchbinder R. The grounded psychometric development and initial validation of the health literacy questionnaire (HLQ). BMC Public Health. 2013;13(1):658. 23855504 10.1186/1471-2458-13-658 PMC3718659

[9] Jessup RL, Osborne RH, Beauchamp A, Bourne A, Buchbinder R. Differences in health literacy profiles of patients admitted to a public and a private hospital in Melbourne, Australia. BMC Health Serv Res. 2018;18(1):134. 29471836 10.1186/s12913-018-2921-4 PMC5824469

[10] Morris RL, Soh S-E, Hill KD, Buchbinder R, Lowthian JA, Redfern J, et al. Measurement properties of the health literacy questionnaire (HLQ) among older adults who present to the emergency department after a fall: a Rasch analysis. BMC Health Serv Res. 2017;17(1):605. 28851344 10.1186/s12913-017-2520-9 PMC5575841

[11] Australian Bureau of Statistics. National Health Survey: Health literacy. 2018. Accessed from https://www.abs.gov.au/statistics/health/health-conditions-and-risks/national-health-survey-health-literacy/latest-release

[12] Simpson RM, Knowles E, O’Cathain A. Health literacy levels of British adults: a cross-sectional survey using two domains of the health literacy questionnaire (HLQ). BMC Public Health. 2020;20(1):1819. 33256670 10.1186/s12889-020-09727-w PMC7708169

[13] Rademakers J, Waverijn G, Rijken M, Osborne R, Heijmans M. Towards a comprehensive, person-centred assessment of health literacy: translation, cultural adaptation and psychometric test of the Dutch health literacy questionnaire. BMC Public Health. 2020;20(1):1850. 33267834 10.1186/s12889-020-09963-0 PMC7709439

[14] Maindal HT, Kayser L, Norgaard O, Bo A, Elsworth GR, Osborne RH. Cultural adaptation and validation of the health literacy questionnaire (HLQ): robust nine-dimension Danish language confirmatory factor model. SpringerPlus. 2016;5(1):1232. 27536516 10.1186/s40064-016-2887-9 PMC4971008

[15] Nolte S, Osborne RH, Dwinger S, Elsworth GR, Conrad ML, Rose M, et al. German translation, cultural adaptation, and validation of the health literacy questionnaire (HLQ). PLoS ONE. 2017;12(2):e0172340. 28234987 10.1371/journal.pone.0172340 PMC5325258

[16] Saleem A, Steadman KJ, Osborne RH, La Caze A. Translating and validating the health literacy questionnaire into Urdu: a robust nine-dimension confirmatory factor model. Health Promot Int. 2021;36(5):1219–30. 33370429 10.1093/heapro/daaa149

[17] Kolarcik P, Cepova E, Madarasova Geckova A, Elsworth GR, Batterham RW, Osborne RH. Structural properties and psychometric improvements of the health literacy questionnaire in a Slovak population. Int J Public Health. 2017;62(5):591–604. 28258403 10.1007/s00038-017-0945-x

[18] Wahl AK, Hermansen Å, Osborne RH, Larsen MH. A validation study of the Norwegian version of the health literacy questionnaire: a robust nine-dimension factor model. Scand J Public Health. 2021;49(4):471–8. 32508258 10.1177/1403494820926428 PMC8135233

[19] Boateng MA, Agyei-Baffour P, Angel S, Enemark U. Translation, cultural adaptation and psychometric properties of the Ghanaian language (akan; Asante Twi) version of the health literacy questionnaire. BMC Health Serv Res. 2020;20(1):1064. 33228648 10.1186/s12913-020-05932-w PMC7684925

[20] Rababah JA, Al-Hammouri MM, Aldalaykeh M. Validation and measurement invariance of the Arabic Health literacy questionnaire. Heliyon. 2022;8(4):e09301. 35497048 10.1016/j.heliyon.2022.e09301 PMC9043993

[21] Huang Y, Ruan T, Yi Q, Wang T, Guo Z. The health literacy questionnaire among the aged in Changsha, China: confirmatory factor analysis. BMC Public Health. 2019;19(1):1220. 31484512 10.1186/s12889-019-7563-x PMC6727331

[22] Budhathoki SS, Hawkins M, Elsworth G, Fahey MT, Thapa J, Karki S et al. Use of the English health literacy questionnaire (HLQ) with health science university students in Nepal: a validity testing study. Int J Environ Res Public Health. 2022;19(6). 10.3390/ijerph19063241 PMC8953246 35328928

[23] Debussche X, Lenclume V, Balcou-Debussche M, Alakian D, Sokolowsky C, Ballet D, et al. Characterisation of health literacy strengths and weaknesses among people at metabolic and cardiovascular risk: validity testing of the health literacy questionnaire. SAGE open Med. 2018;6:2050312118801250. 30319778 10.1177/2050312118801250 PMC6154264

[24] Arsenović S, Oyewole O, Trajković G, Osborne RH, Wiltshire-Fletcher M, Gazibara T et al. Linguistic adaptation and psychometric properties of the health literacy questionnaire in Serbian language among people with chronic diseases. Chronic illn. 2022:17423953221102630. 10.1177/17423953221102630 35581691

[25] Hawkins M, Gill SD, Batterham R, Elsworth GR, Osborne RH. The health literacy questionnaire (HLQ) at the patient-clinician interface: a qualitative study of what patients and clinicians mean by their HLQ scores. BMC Health Serv Res. 2017;17(1):309. 28449680 10.1186/s12913-017-2254-8 PMC5408483

[26] Dodson S, Good S, Osborne RH. Health literacy toolkit for low and middle-income countries: a series of information sheets to empower communities and strength health systems. New Delhi: Worth Health Organization, Regional Office for South-East Asia, 2015. 2015.

[27] Dinh TTH, Bonner A. Exploring the relationships between health literacy, social support, self-efficacy and self-management in adults with multiple chronic diseases. BMC Health Serv Res. 2023;23(1):923. 37649013 10.1186/s12913-023-09907-5 PMC10466814

[28] Dinh HTT, Nguyen NT, Bonner A. Health literacy profiles of adults with multiple chronic diseases: a cross-sectional study using the health literacy questionnaire. Nurs Health Sci. 2020;22(4):1153–60. 33034404 10.1111/nhs.12785

[29] Dinh HTT, Nguyen NT, Bonner A. Healthcare systems and professionals are key to improving health literacy in chronic kidney disease. J Ren Care. 2022;48(1):4–13. 34291578 10.1111/jorc.12395

[30] Shahid R, Shoker M, Chu LM, Frehlick R, Ward H, Pahwa P. Impact of low health literacy on patients’ health outcomes: a multicenter cohort study. BMC Health Serv Res. 2022;22(1):1148. 36096793 10.1186/s12913-022-08527-9 PMC9465902

[31] Muscat DM, Cvejic E, Bell K, Smith J, Morris GM, Jansen J, et al. The impact of health literacy on psychosocial and behavioural outcomes among people at low risk of cardiovascular disease. Prev Med. 2022;156:106980. 35122835 10.1016/j.ypmed.2022.106980

[32] Kline RB. Principles and practice of structural equation modeling. Guilford; 2023.

[33] Charlson M, Szatrowski TP, Peterson J, Gold J. Validation of a combined comorbidity index. J Clin Epidemiol. 1994;47(11):1245–51. 7722560 10.1016/0895-4356(94)90129-5

[34] Hawkins M, Cheng C, Elsworth GR, Osborne RH. Translation method is validity evidence for construct equivalence: analysis of secondary data routinely collected during translations of the health literacy questionnaire (HLQ). BMC Med Res Methodol. 2020;20(1):130. 32456680 10.1186/s12874-020-00962-8 PMC7249296

[35] JASP team. (2024). JSAP (version 0.18.3) [Computer software].

[36] West SG, Taylor AB, Wu W. Model fit and model selection in structural equation modeling. Handb Struct Equation Model. 2012;1:209–31.

[37] Smith G, Lui SF, Kalantar-Zadeh K, Bonner A. The shift from individual to organizational health literacy: implications for kidney healthcare leaders and clinicians. Nephron. 2023;148(5):1–1. 38109858 10.1159/000534073

[38] Elsworth GR, Nolte S, Cheng C, Hawkins M, Osborne RH. Modelling variance in the multidimensional health literacy questionnaire: does a General Health Literacy factor account for observed interscale correlations? SAGE Open Med. 2022;10:1–18. 10.1177/20503121221124771 PMC9511310 36172568

