# Supplementary material for: Psychometric properties of the health literacy questionnaire tested in Vietnamese adults with chronic diseases
Source: BMC Public Health. 2025 Jan 6;25:44. doi: 10.1186/s12889-024-21156-7 (PMC11702074; doi:10.1186/s12889-024-21156-7)
Supplement: Supplementary file 1 — Supplementary Material 1 [file 12889_2024_21156_MOESM1_ESM.docx]

***Supplemental Table 1a. Models exploring factors influencing to health literacy domains***

|  | 1. Feeling understood and supported by HCPs | | | 2. Having sufficient information to manage my health | | | 3. Actively managing my health | | | 4. Social support for health | | | 5. Appraisal of health information | | |
| --- | --- | --- | --- | --- | --- | --- | --- | --- | --- | --- | --- | --- | --- | --- | --- |
| Factors | B | 95% CI | Sig. | B | 95% CI | Sig. | B | 95% CI | Sig. | B | 95% CI | Sig. | B | 95% CI | Sig. |
| Intercept | 2.63 | 2.50, 2.76 | **< 0.001** | 2.83 | 2.72, 2.96 | **< 0.001** | 3.04 | 2.96, 3.12 | **< 0.001** | 3.24 | 3.12, 3.35 | **< 0.001** | 2.89 | 2.76, 3.01 | **< 0.001** |
| **Education (**≤ High school) | -0.20 | -0.31, -0.09 | **< 0.001** |  |  |  | -0.12 | -0.21, -0.03 | **0.01** |  |  |  | -0.22 | -0.32, -0.12 | **< 0.001** |
| **Living in urban** | 0.10 | 0.02, 0.19 | **0.01** | 0.10 | 0.02, 0.18 | **0.01** |  |  |  | 0.11 | 0.05, 0.18 | **0.001** |  |  |  |
| **Main carer (**Spouse/Child) | 0.12 | 0.02, 0.23 | **0.02** |  |  |  |  |  |  | 0.19 | 0.11, 0.27 | **< 0.001** |  |  |  |
| **Monthly income** |  |  |  |  |  |  |  |  |  |  |  |  |  |  |  |
| ≤ 5 million |  |  |  | -0.24 | -0.36, -0.11 | **< 0.001** |  |  |  | -0.25 | -0.35, -0.15 | **< 0.001** | -0.15 | -0.27, -0.03 | **0.02** |
| 5-10 million |  |  |  | -0.09 | -0.23, 0.05 | 0.20 |  |  |  | -0.17 | -0.28, -0.06 | **0.003** | -0.05 | -0.19, 0.08 | 0.45 |
| **ACCI (< 6)** |  |  |  |  |  |  |  |  |  |  |  |  | 0.09 | 0.006, 0.17 | **0.04** |

Note. Referent groups: Education: A degree or higher education, Residence: living in rural/remote area, Main support person: others (not spouse or children), Family income ≥ 10 million VND per month, ACCI (Age-adjusted Charlson Comorbidity Index): ≥ 6, B: adjusted mean difference, CI: Confidence Interval. HCPs: healthcare providers.

***Supplemental Table 1b. Models exploring factors influencing to health literacy domains (continued)***

|  | 6. Ability to actively engage with HCPs | | | 7. Navigating the healthcare system | | | 8. Ability to find good health information | | | 9. Understanding health information well enough to know what to do | | |
| --- | --- | --- | --- | --- | --- | --- | --- | --- | --- | --- | --- | --- |
| Factors | B | 95% CI | Sig. | B | 95% CI | Sig. | B | 95% CI | Sig. | B | 95% CI | Sig. |
| Intercept | 3.98 | 3.82, 4.13 | **<0.001** | 3.98 | 3.82, 4.13 | **< 0.001** | 3.75 | 3.55, 3.94 | **< 0.001** | 3.68 | 3.50, 3.87 | **< 0.001** |
| **Age** (< 65 yo) | 0.12 | 0.03, 0.22 | **0.007** | 0.12 | 0.03, 0.21 | **0.009** | 0.15 | 0.02, 0.28 | **0.02** | 0.29 | 0.18, 0.40 | **< 0.001** |
| **Education (**≤ High school) | -0.20 | -0.33, -0.08 | **0.001** | -0.29 | -0.41, -0.16 | **< 0.001** | -0.33 | -0.49, -0.17 | **< 0.001** | -0.31 | -0.46, -0.17 | **< 0.001** |
| **Monthly income** |  |  |  |  |  |  |  |  |  |  |  |  |
| ≤ 5 million | -0.29 | -0.44, -0.15 | **< 0.001** | -0.28 | -0.42, -0.13 | **< 0.001** | -0.44 | -0.62, -0.25 | **< 0.001** | -0.38 | -0.55, -0.20 | **< 0.001** |
| 5-10 million | -0.12 | -0.28, 0.04 | 0.15 | -0.09 | -0.26, 0.07 | 0.26 | -0.19 | -0.39, 0.02 | 0.07 | -0.17 | -0.36, 0.02 | 0.08 |
| **ACCI (< 6)** |  |  |  |  |  |  | 0.22 | 0.08, 0.37 | **0.002** |  |  |  |

Note. Referent groups: Age ≥ 65, Education: A degree or higher, Family income ≥ 10 million VND per month, ACCI (Age-adjusted Charlson Comorbidity Index): ≥ 6, B: adjusted mean difference, CI: Confidence Interval. HCPs: healthcare providers.

***Supplemental Table 2a. Health literacy domains scores and effect sizes in different demographic groups (n = 600)***

| **Variables** | **1.Feeling understood and supported by HCPs** | **2.Having sufficient information to manage my health** | **3.Actively managing health** | **4.Social support for health** | **5. Appraisal of health information** |
| --- | --- | --- | --- | --- | --- |
| **Age** § | **P = 0.007** | p = 0.75 | **P = 0.02** | **P= 0.03** | P= 0.35 |
| < 65 years (n = 307) | 2.55 (0.49) | 2.70 (0.48) | 2.90 (0.4) | 3.21 (0.40) | 2.64 (0.44) |
| ≥ 65 years (n = 293) | 2.67 (0.52) | 2.72 (0.53) | 2.99 (0.48) | 3.28 (0.44) | 2.60 (0.53) |
| *Effect size* | *0.24* | *0.04* | *0.20* | *0.17* | *0.08* |
| **Gender** § | P = 0.47 | P= 0.84 | P = 0.13 | P = 0.87 | P = 0.26 |
| Male (n = 326) | 2.62 (0.52) | 2.71 (0.51) | 2.92 (0.44) | 3.24 (0.42) | 2.64 (0.48) |
| Female (n = 274) | 2.59 (0.49) | 2.71 (0.50) | 2.97 (0.45) | 3.25 (0.43) | 2.60 (0.49) |
| *Effect size* | *0.06* | *0* | *0.11* | *0.02* | *0.08* |
| **Marital status** § | P = 0.51 | P = 0.28 | P = 0.46 | P = 0.91 | P = 0.75 |
| Married (n = 502) | 2.61 (0.51) | 2.70 (0.51) | 2.94 (0.44) | 3.24 (0.42) | 2.62 (0.48) |
| Divorced/Widowed/Single (n = 98) | 2.58 (0.53) | 2.76 (0.47) | 2.97 (0.45) | 3.24 (0.42) | 2.64 (0.47) |
| *Effect size* | *0.06* | *0.12* | *0.07* | *0* | *0.04* |
| **Level of education** § | **P < 0.001** | **P = 0.001** | **P = 0.01** | **P = 0.02** | **P < 0001** |
| Up to year 12 (n = 494) | 2.57 (0.49) | 2.68 (0.50) | 2.92 (0.44) | 3.22 (0.42) | 2.58 (0.48) |
| VET/University/Higher degree (n = 106) | 2.79 (0.55) | 2.86 (0.48) | 3.04 (0.42) | 3.33 (0.42) | 2.83 (0.48) |
| *Effect size* | *0.44* | *0.36* | *0.27* | *0.26* | *0.52* |
| **Residential area** § | **p< 0.001** | **P= 0.001** | P= 0.10 | **p< 0.001** | **P= 0.002** |
| City/urban (n = 262) | 2.69 (0.54) | 2.79 (0.48) | 2.98 (0.48) | 3.34 (0.45) | 2.69 (0.51) |
| Rural, remote, mountainous (n = 338) | 2.54 (0.48) | 2.65 (0.51) | 2.92 (0.41) | 3.17 (0.39) | 2.57 (0.46) |
| *Effect size* | *0.29* | *0.28* | *0.13* | *0.40* | *0.25* |
| **Monthly income**¶ | **P = 0.03** | **P = < 0.001** | P= 0.48 | **P < 0.001** | **P < 0.001** |
| ≤ 5 million VND (n = 395) | 2.57 (0.46) | 2.64 (0.50) | 2.93 (0.43) | 3.19 (0.40) | 2.56 (0.48) |
| 5 -10 million VND (n = 128) | 2.64 (0.61) | 2.81 (0.49) | 2.98 (0.47) | 3.29 (0.42) | 2.69 (0.51) |
| ≥ 10 million VND (n = 77) | 2.73 (0.56) | 2.90 (0.45) | 2.95 (0.43) | 3.45 (0.44) | 2.80 (0.44) |
| *Effect size* |  |  |  |  |  |
| ≤ 5 million VND vs ≥ 10 million VND | *0.33* | *0.53* | *0.05* | *0.64* | *0.51* |
| 5-10 million VND vs ≥ 10 million VND | *0.15* | *0.19* | *0.07* | *0.37* | *0.23* |
| **Main support person** § | **P= 0.02** | P= 0.56 | P= 0.08 | **P < 0.001** | P = 0.75 |
| Spouse/children (n = 488) | 2.63 (0.51) | 2.72 (0.51) | 2.96 (0.44) | 3.28 (0.40) | 2.63 (0.50) |
| Other people (n = 112) | 2.51 (0.51) | 2.69 (0.48) | 2.88 (0.44) | 3.08 (0.46) | 2.61 (0.41) |
| *Effect size* | *0.23* | *0.06* | *0.18* | *0.48* | *0.04* |
| **ACCI** § | P= 0.50 | P= 0.55 | P= 0.46 | P= 0.29 | **P= 0.03** |
| Mild/moderate (n = 172) | 2.59 (0.54) | 2.73 (0.47) | 2.92 (0.43) | 3.27 (0.43) | 2.69 (0.45) |
| Severe (n = 428) | 2.62 (0.50) | 2.70 (0.52) | 2.95 (0.45) | 3.23 (0.42) | 2.59 (0.49) |
| *Effect size* | *0.06* | *0.06* | *0.07* | *0.09* | *0.21* |

Note: ACCI: Age-adjusted Charlson Comorbidity Index, HCPs: healthcare providers, VET: vocational education and training, VND: Vietnam dong, ¶ ANOVA test, § independent t-test.

***Supplemental Table 2b*. *Health literacy domains scores and effect size in different demographic groups (n = 600) (continues…)***

| **Variables** | **6.Ability to actively engage with HCPs** | **7.Navigating the healthcare system** | **8.Ability to find good health information** | **9. Understanding health information well enough to know what to do** |
| --- | --- | --- | --- | --- |
| **Age** § | **P = 0.002** | **P = 0.003** | **P < 0.001** | **P = 0.02** |
| < 65 years (n = 307) | 3.73 (0.55) | 3.68 (0.55) | 3.43 (0.71) | 3.45 (0.66) |
| ≥ 65 years (n = 293) | 3.58 (0.62) | 3.53 (0.63) | 3.14 (0.79) | 3.13 (0.74) |
| *Effect size* | *0.26* | *0.25* | *0.39* | *0.46* |
| **Gender** § | P = 0.26 | P = 0.63 | P = 0.10 | **P = 0.03** |
| Male (n = 326) | 3.67 (0.59) | 3.64 (0.58) | 3.35 (0.75) | 3.36 (0.68) |
| Female (n = 274) | 3.64 (0.58) | 3.56 (0.61) | 3.21 (0.77) | 3.22 (0.75) |
| *Effect size* | *0.05* | *0.13* | *0.18* | *0.19* |
| **Marital status** § | P = 0.62 | P = 0.06 | P = 0.09 | P = 0.19 |
| Married (n = 502) | 3.66 (0.59) | 3.63 (0.58) | 3.31 (0.75) | 3.31 (0.71) |
| Divorced/Widowed/Single (n = 98) | 3.63 (0.58) | 3.50 (0.67) | 3.19 (0.81) | 3.21 (0.73) |
| *Effect size* | *0.05* | *0.22* | *0.16* | *0.14* |
| **Level of education** § | **P < 0.001** | **P < 0.001** | **P < 0.001** | **P < 0.001** |
| Up to year 12 (n = 494) | 3.6 (0.60) | 3.54 (0.61) | 3.21 (0.77) | 3.22 (0.72) |
| VET/University/Higher degree (n = 106) | 3.89 (0.45) | 3.91 (0.43) | 3.65 (0.63) | 3.63 (0.56) |
| *Effect size* | *0.50* | *0.63* | *0.59* | *0.59* |
| **Residential area** § | P = 0.12 | **P = 0.02** | **P = 0.04** | P = 0.85 |
| City/urban (n = 262) | 3.70 (0.61) | 3.67 (0.61) | 3.36 (0.79) | 3.30 (0.73) |
| Rural, remote, mountainous (n = 338) | 3.62 (0.57) | 3.56 (0.58) | 3.23 (0.74) | 3.29 (0.70) |
| *Effect size* | *0.14* | *0.18* | *0.17* | *0.01* |
|  |  |  |  |  |
| **Monthly income**¶ | **P < 0.001** | **P < 0.001** | **P < 0.001** | **P < 0.001** |
| ≤ 5 million VND (n = 395) | 3.56 (0.62) | 3.51 (0.62) | 3.14 (0.76) | 3.17 (0.71) |
| 5 -10 million VND (n = 128) | 3.77 (0.51) | 3.73 (0.52) | 3.45 (0.74) | 3.42 (0.66) |
| ≥ 10 million VND (n = 77) | 3.96 (0.39) | 3.91 (0.42) | 3.77 (0.59) | 3.72 (0.62) |
| *Effect size* |  |  |  |  |
|  |  |  |  |  |
| ≤ 5 million VND vs ≥ 10 million VND | *0.68* | *0.67* | *0.86* | *0.79* |
| 5-10 million VND vs ≥ 10 million VND | *0.40* | *0.37* | *0.47* | *0.46* |
| **Main support person** § | P = 0.92 | P= 0.55 | P= 0.08 | **P= 0.01** |
| Spouse/children (n= 488) | 3.65 (0.58) | 3.61 (0.59) | 3.26 (0.76) | 3.26 (0.70) |
| Other people (n = 112) | 3.66 (0.61) | 3.58 (0.62) | 3.40 (0.76) | 3.45 (0.74) |
| *Effect size* | *0.02* | *0.05* | *0.18* | *0.27* |
| **ACCI** § | **P= 0.005** | **P= 0.009** | **P < 0.001** | **P < 0.001** |
| Mild/moderate (n = 172) | 3.76 (0.56) | 3.71 (0.57) | 3.53 (0.76) | 3.48 (0.71) |
| Severe (n = 428) | 3.61 (0.59) | 3.57 (0.60) | 3.19 (0.74) | 3.22 (0.70) |
| *Effect size* | *0.26* | *0.24* | *0.45* | *0.37* |

*Note.* ACCI: Age-adjusted Charlson Comorbidity Index, HCPs: healthcare providers, VET: vocational education and training, VND: Vietnam dong, ¶ ANOVA test, § independent t-test.

**Supplement Table 3. Factor loadings of items in the 9-factor CFA model of Health Literacy Questionnaire**

| Scale | Factor loading |
| --- | --- |
| HLQ 1. Healthcare provider support |  |
| HLQ1.2 | 0.92 |
| HLQ1.8 | 0.89 |
| HLQ1.17 | 0.81 |
| HLQ1.22 | 0.80 |
| HLQ 2. Having sufficient information |  |
| HLQ1.1 | .77 |
| HLQ1.10 | 0.90 |
| HLQ1.14 | 0.86 |
| HLQ1.23 | 0.83 |
| HLQ 3. Actively managing my health |  |
| HLQ1.6 | 0.77 |
| HLQ1.9 | 0.87 |
| HLQ1.13 | 0.81 |
| HLQ1.18 | 0.85 |
| HLQ1.21 | 0.74 |
| HLQ4. Social support for health |  |
| HLQ1.3 | 0.84 |
| HLQ1.5 | 0.79 |
| HLQ1.11 | 0.76 |
| HLQ1.15 | 0.67 |
| HLQ1.19 | 0.88 |
| HLQ 5. Appraisal of health information |  |
| HLQ 1.4 | 0.85 |
| HLQ 1.7 | 0.82 |
| HLQ 1.12 | 0.87 |
| HLQ 1.16 | 0.85 |
| HLQ 1.20 | 0.74 |
| HLQ 6. Ability to actively engage with HCPs |  |
| HLQ 2.2 | 0.79 |
| HLQ 2.4 | 0.82 |
| HLQ 2.7 | 0.89 |
| HLQ 2.15 | 0.88 |
| HLQ 2.20 | 0.89 |
| HLQ 7. Navigating the healthcare system |  |
| HLQ 2.1 | 0.67 |
| HLQ 2.8 | 0.83 |
| HLQ 2.11 | 0.82 |
| HLQ 2.13 | 0.88 |
| HLQ 2.16 | 0.83 |
| HLQ 2.19 | 0.89 |
| HLQ 8. Ability to find good health information |  |
| HLQ 2.3 | 0.85 |
| HLQ 2.6 | 0.88 |
| HLQ 2.10 | 0.82 |
| HLQ 2.14 | 0.87 |
| HLQ 2.18 | 0.91 |
| HLQ 9. Understand health information |  |
| HLQ 2.5 | 0.86 |
| HLQ 2.9 | 0.78 |
| HLQ 2.12 | 0.91 |
| HLQ 2.17 | 0.89 |
| HLQ 2.21 | 0.87 |
| Fit indexes: X2 = 5537.4, 866 df, p < 0.001, CFI = 0.98, NFI = 0.98, RMSEA = 0.09, 90% CI (0.093, 0.097), PCLOSE < 0.001 | |

**Supplemental Table 4: Inter-factor correlation coefficients between Health Literacy Questionnaire scales (from the 9-factor CFA model)**

| Scale | HLQ 1. Healthcare provider support | HLQ 2. Having sufficient information | HLQ 3. Actively managing my health | HLQ 4. Social support for health | HLQ 5. Appraisal of health information | HLQ 6. Ability to actively engage with HCPs | HLQ 7. Navigating the healthcare system | HLQ 8. Ability to find good health information |
| --- | --- | --- | --- | --- | --- | --- | --- | --- |
| HLQ 2. Having sufficient information | 0.54 | 1 |  |  |  |  |  |  |
| HLQ 3. Actively managing my health | 0.48 | 0.67 | 1 |  |  |  |  |  |
| HLQ 4. Social support for health | 0.46 | 0.58 | 0.58 | 1 |  |  |  |  |
| HLQ 5. Appraisal of health information | 0.61 | 0.79 | 0.72 | 0.42 | 1 |  |  |  |
| HLQ 6. Ability to actively engage with HCPs | 0.44 | 0.55 | 0.52 | 0.38 | 0.61 | 1 |  |  |
| HLQ 7. Navigating the healthcare system | 0.46 | 0.53 | 0.51 | 0.33 | 0.61 | 0.96 | 1 |  |
| HLQ 8. Ability to find good health information | 0.39 | 0.57 | 0.46 | 0.32 | 0.67 | 0.80 | 0.83 | 1 |
| HLQ9. Understand health information | 0.30 | 0.47 | 0.43 | 0.22 | 0.53 | 0.76 | 0.76 | 0.90 |

Note. All correlation coefficients were significant at p < 0.001.
